# Yulendji Bunwurrung Walagunh With Western Science: Ecological Insights, Emerging Research Questions and Future Directions From Bunurong Stories of Marine Mammals

**DOI:** 10.1002/ece3.74104

**Published:** 2026-07-29

**Authors:** Jemima Beddoe, Gail Kunwarra Dawson, Jeff Shimeta, Kate Robb

**Affiliations:** ^1^ School of Science RMIT University Melbourne Victoria Australia; ^2^ Marine Mammal Foundation Hampton East Victoria Australia; ^3^ Eliza Nowen Apical Ancestor Group, Bunurong Community Melbourne Victoria Australia

**Keywords:** Indigenous Knowledge, marine conservation, marine mammals, western science

## Abstract

Indigenous Knowledge systems contain deep, place‐based ecological insights developed over generations, yet they remain underrepresented in mainstream biodiversity assessments and conservation planning. In Australia, Indigenous peoples have maintained enduring relationships with **Warrinh** (sea/ocean) for at least 65,000 years, generating rich knowledge of marine ecosystems and species. This study explores **yulendji Bunwurrung** (Bunurong knowledge) related to marine mammal ecology in Victoria, Australia. Five Stories shared by a Bunurong Elder describe Bunwurrung women's connection to Sea Country and their Knowledge of marine mammals. A thematic analysis was used to identify key ecological themes within the Stories, which were then integrated with contemporary scientific literature to explore areas of convergence and identify emerging research questions. The Stories describe species occurrences, habitat use, seasonal movements, interspecific relationships and historical conditions that aligned with, and in some cases extended beyond, the temporal scope of contemporary scientific records. Several Stories also highlighted areas where scientific understanding remains understudied, including marine mammal movement patterns, habitat use and species interactions. The findings emphasise the importance of Indigenous‐led knowledge as a source of ecological understanding and hypothesis generation, while emphasising the importance of respectfully integrating multiple knowledge systems to support marine research, conservation and management.

## Graphical abstract

Indigenous Knowledge systems contain deep, place‐based ecological insights developed over generations but remain underrepresented in biodiversity assessments and conservation planning. This study explores yulendji Bunwurrung (Bunurong knowledge) through five Stories shared by a Bunurong Elder, using thematic analysis and comparison with contemporary scientific literature to examine marine mammal ecology in Victoria, Australia. The Stories revealed substantial areas of convergence with contemporary scientific literature while also contributing ecological observations that extend beyond the current scientific record, demonstrating the value of Indigenous‐led knowledge for generating research questions and supporting marine conservation through respectful knowledge integration.

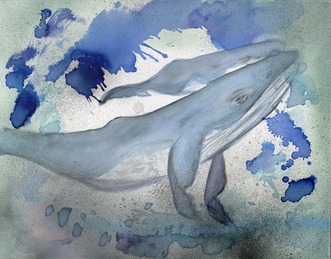

## 1 Introduction

Australia is home to the longest living Indigenous culture on earth and also contains the third largest marine territory in the world (Hedge et al. 2020). For at least 65,000 years, Indigenous peoples have occupied the Australian continent (Clarkson et al. 2017; Rist et al. 2019). For Indigenous peoples in Australia, ‘Country’ encompasses everything: ‘Rock, tree, river, hill, animal, human – all were formed of the same substance by the Ancestors who continue to live in land, water, sky … Country is family, culture, identity, Country is self’ (Kwaymullina 2004, 12), and ‘Within this, one Entity should not be raised above another, as these live in close relationship with one another. So people are no more or less important than other Entities’ (Martin‐Booran Mirraboopa 2003, 207). The terms ‘Sea Country’ and ‘Saltwater Country’ are used to describe the coastal, island and marine environments—including beaches, seagrass beds, reefs, estuaries and bays and coastal wetlands—to which Indigenous cultural rights, Custodianship, authority and complex biocultural systems apply (Rist et al. 2019; Ward et al. 2018). This connection does not only relate to the coastlines of the present day but also relates to the sea‐level changes seen throughout many millennia (Morphy et al. 2020), which approximately 22,000 years ago saw coastlines up to 300 km further offshore and the relative sea levels ~130 m below present (Ward et al. 2018).

Indigenous Ecological Knowledge (IEK) is used here as defined by Berkes (2008); ‘… cumulative body of Knowledge, practice and belief evolving by adaptive processes and handed down through generations by cultural transmission about the relationship of living beings (including humans) with one another and with their environment’. IEK is often overlooked and deemed incompatible with dominant western scientific systems, despite its ability to provide vital ecological insights, especially for culturally significant species (Campbell et al. 2022; Skroblin et al. 2021). Culturally significant species (or entities) are species or ecological communities (landscape/seascapes) which have cultural value to Indigenous peoples and are important for their relationship with and adaptation to Country (Goolmeer and van Leeuwen 2023; Goolmeer et al. 2022).

Indigenous Knowledge exists largely in oral forms, typically held by Indigenous Elders and Custodians. Storytelling is one of the main forms of transmitting and sharing Knowledge in Indigenous societies (Bessarab and Ng'andu 2010). Holt and Perry (2023) define Indigenous Storytelling as a cultural conduit to impart and share Knowledge as well as understanding and formulating new knowledge. Indigenous Stories and Storytelling honour the holistic nature of the environment; they do not view ecosystems as a mechanism distinct from people and feeling, but rather as an integrated system that is based upon reciprocal human‐animal relationships (Lyver et al. 2009), as well as relationships between other animals and the environment, and humans and the environment (Gibbs et al. 2025). This knowledge can be valuable for natural resource assessments and local ecological understanding (Fernández‐Llamazares and Cabeza 2018), with great value in partnerships that seek to integrate and understand the differences and complementarities of each knowledge system (Jackson et al. 2014; Woodward and Marrfurra McTaggart 2016; Muller 2014). With technological developments, Traditional Custodians from across the world have evolved their preservation of Cultural Knowledge, language and history, with the development of Indigenous digital libraries (Shiri et al. 2022). These digital media projects offer the potential for information and knowledge to be preserved and passed on to future generations (Edmonds 2014a), by integrating storytelling practices for a more dynamic approach to sharing.

Due to the forced assimilation of Indigenous people, including the transgenerational effects of the Stolen Generations in Australia, some cultural and spiritual Knowledge has been lost (Cameron 2022). While some western scientists have attempted to engage with Indigenous Peoples and their Knowledge, Campbell et al. (2022) highlight concerns that these engagements can be extractive, while Ens et al. (2015) caution against the ‘scientising’ and ‘distilling’ of IEK to fit western scientific frameworks. Many regions that have been subjected to European colonisations still validate IEK according to conventional scientific paradigms, which further perpetuates colonial power imbalances (Thompson et al. 2019). This has been documented to be due to the view that IEK is primitive, folkloric, anecdotal, unscientific and lacking in rigour and objectivity (Berkes 2012; Ens et al. 2015; Gibbs et al. 2025; Jessen et al. 2022; Knopf 2015). Regardless of the fact that Indigenous peoples are often the first to observe changes in their Sea Country, such as changes in biodiversity, declines in species abundance or changes in population composition (Ens et al. 2015; McLean et al. 2023), the inclusion of such knowledge is not widely accepted in baseline ecological assessments. These knowledge systems have been developed over centuries, gained from experience and adaptation to local culture and the environment (Mulalap et al. 2020), and can provide a more accurate baseline measure of place‐specific and/or species‐specific conditions (Cameron 2022).

Collaborative projects have shown the depth of knowledge held by Indigenous communities and the extent to which IEK can contribute to a range of different sectors across both terrestrial and marine environments. IEK has contributed to biodiversity assessments and ecological understanding through participatory mapping in Chile with Mapuche, which documented detailed Indigenous territorial dimensions (Hirt 2012), and through coproduction of knowledge regarding the **Iqalukjuaq** (Greenland shark) with the Inuit community of Pangnirtung in southern Baffin Island (Idrobo and Berkes 2012). Similarly, the weaving of Māori knowledge and western science (WS) has helped characterise biodiversity hotspots in Aotearoa and reconstruct historical baselines of marine mammal occurrence (Brough et al. 2025). IEK has also informed species conservation and recovery. For example, the Martu (traditional custodians of the Martu Native Title Determination Area in Western Australia) contributed knowledge to the conservation and recovery of the **mankarr** (greater bilby) (Skroblin et al. 2021), while **mātauranga Māori** (Māori marine knowledge systems) has been incorporated to inform marine mapping and management to support conservation planning and environmental stewardship (Paul‐Burke et al. 2020). Furthermore, IEK has been integrated with WS in regard to environmental governance and the recognition of culturally significant species. Indigenous‐led and collaboratively governed conservation initiatives, such as the Dhimurru Indigenous Protected Area in the Northern Territory, demonstrate how Traditional Owners can play a central role in environmental management (Rist et al. 2019). Additionally, following the formal identification of *Tursiops australis* in south‐eastern Australia, collaboration between researchers and local Boonwurrung Elders and the Victorian Aboriginal Corporation for Languages contributed to formally naming the endemic dolphin, **Burrunan** (dolphin—Boonwurrung, Woiwurrung and Taungurung Languages of the Kulin Nation) dolphin (Charlton‐Robb et al. 2011), recognising both the scientific distinctiveness and cultural significance of the species.

While the above studies, and the collaborations they represent, demonstrate how IEK can contribute to environmental governance and management approaches that support healthy marine ecosystems (Parsons et al. 2021), there are also international and national initiatives underway to address inequity and promote self‐determination. The right of Indigenous Peoples in international law and policy was outlined in the 2007 United Nations Declaration on the Rights of Indigenous Peoples (UNIDRIP). Although Australia was initially one of four nations to oppose the declaration, it formally endorsed UNIDRIP in 2009 (Conrad et al. 2025). More recently, Conrad et al. (2025) developed the Blue Peacebuilding Scorecard (BPS) to evaluate Indigenous marine governance programs in Australia, New Zealand and Canada, following the endorsement of UNIDRIP. With the implementation of three national programs (‘Sea Country Indigenous Protected Areas Program, Indigenous Ranger Programs and Aboriginal Water Entitlements Program’ n.d.), Australia showed strengths in the areas of justice and impact, whereas areas of improvement included legacy, data sovereignty and long‐term financial support. The revitalisation of language, equitable data practices and fostering collaborative approaches to enhance marine governance were identified as priorities for strengthening Indigenous participation in marine conservation management.

Despite the increasing recognition of IEK within marine conservation, there remains a need for place‐based studies that document and apply local Indigenous Knowledge systems with contemporary environmental research and management. By exploring ecological knowledge embedded within Bunurong Stories, this study contributes to broader national and international efforts to recognise IEK as a complementary and important source of information for conservation planning, biodiversity monitoring and marine governance. Therefore, the aim of this study is to (1) integrate local IEK and WS knowledge of marine mammals, a process described as **Walagunh Baanhbula** (mixing two waters), in the Bunwurrung language, and (2) identify ecological research questions and potential regions for conservation based on **Yulendji Bunwurrung** (Bunurong knowledge). We examine how knowledge embedded within Bunurong Stories relates to species distribution, behaviours and ecological processes in the Victorian marine ecosystem. By bringing these two knowledge systems together, this study provides a more holistic and temporally expansive understanding of marine mammals in Victoria, while also demonstrating the value of knowledge integration for ecological baselines, monitoring and conservation planning.

Aunty Gail Dawson (AGD), a Bunurong Elder, has shared these Stories as the women of the Bunurong are Custodians of **Warrinh Biik** (Sea Country) and the following Stories reflect her Knowledge of marine mammals and relationships with County. It is important to recognise that there is no singular IEK system. Indigenous Knowledge(s) are diverse, context‐specific and shaped by distinct cultures, languages and environments (Stein et al. 2024; Robin et al. 2022). Thus, while examples from other Indigenous communities from across the globe are discussed throughout this manuscript, this Knowledge and the Stories shared here are specific to Bunurong Country. These Stories represent relationships to Sea Country and the marine mammals that are unique to Bunurong peoples and should not be interpreted as representative of IEK elsewhere.

## 2 Methods

### 2.1 Bunurong Territory

**FIGURE 1:**
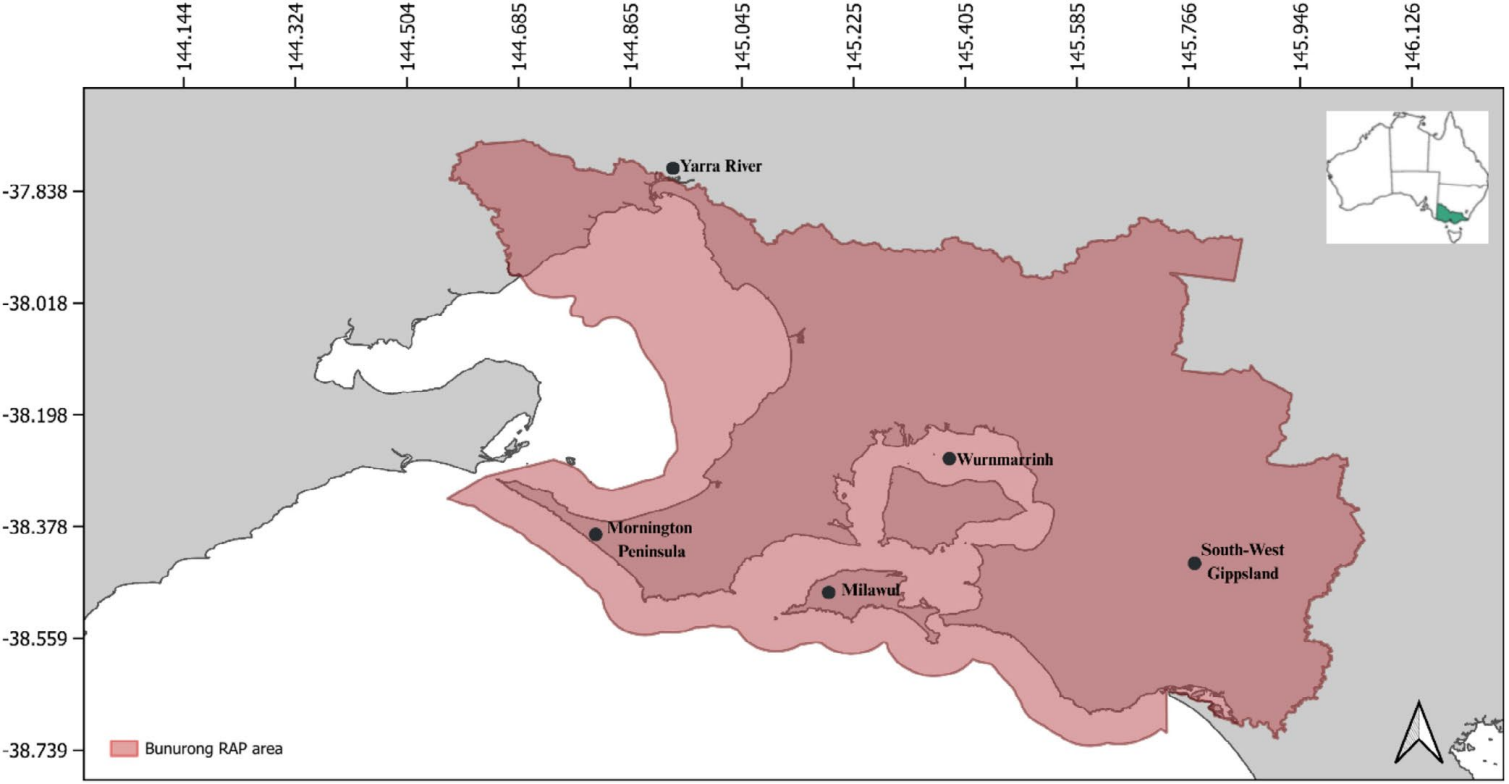
Government‐approved Bunurong Land Council Aboriginal Corporation Registered Aboriginal Party (RAP) area, Victoria, Australia.

The Government‐approved Bunurong territory (Figure 1) stretches from the Mornington Peninsula to Western Port Bay and includes a portion of South‐West Gippsland. However, the Bunurong peoples continue to contest this boundary designation, negotiating for the boundary to represent their connection and Custodianship of Country by extending to the eastern bank of the **Wirribi yaluk** (Werribee), to the south bank of the Yarra River, across to the Dandenong Ranges and all the way south to **Wamum** (Wilsons Promontory). The Bunurong Land Council Aboriginal Corporation is established as the Registered Aboriginal Party (RAP) that represents the Bunurong peoples and is, therefore, a statutory decision‐maker under the *Aboriginal Heritage Act 2006* (Aboriginal Heritage Act 2006 2023).

### 2.2 Project Initiation

The Bunurong Land Council Aboriginal Corporation (BLCAC) was approached by lead researcher JB to take part in this study. This initial contact involved a meeting with BLCAC representatives and authors JB and KR to discuss potential facilitation and exploration of marine mammal Knowledge within this project. Following this meeting, a project plan was developed outlining the proposed topics of interest, potential methods and approaches to data documentation. This plan was shared with BLCAC for feedback, edits and approval.

Once approved, the project plan was circulated within the BLCAC community to invite expressions of interest from potential participants. AGD responded to this invitation and expressed interest in participating in the project and sharing her Knowledge. While the broader project collected data through yarns, or semi‐structured interviews, between JB and BLCAC community members, the information shared during those interviews is not fully included in this study. Following her project interview, AGD approached JB with the wish to share Stories relating to Bunwurrung women's connection to Sea Country and marine mammals.

### 2.3 Gathering Story

AGD wrote the following Stories to represent yulendji Bunwurrung and the connection of Bunwurrung women to Sea Country. These Stories were written by AGD and shared with JB; no edits have been made to the content or language used in these Stories by any of the authors other than AGD. The development of the manuscript was shared between JB and AGD, ensuring the accurate interpretation of the Stories and language.

Following the completion of the Stories, AGD suggested that accompanying artwork could further support the sharing of their significance. In response, Jayde Halls, a Bunurong woman, created five pieces of artwork to pair with each Bunurong Story. Communicating through artwork has been identified as an important means of sharing Knowledge about Sea Country and the management of Country with both the broader community and other Indigenous communities (Zurba and Berkes 2014). Accordingly, the inclusion of artwork formed part of this study's broader commitment to integrating Indigenous Knowledge systems and communication practices into ecological research.

### 2.4 Content Analysis

After the Stories were received, JB undertook a thematic analysis of each Story following the six‐phase framework of Braun and Clarke (2006). Initial stages of the analysis, including familiarisation with the data, generating initial codes and searching for themes, were conducted by JB. The reviewing of themes and defining/naming of themes were then undertaken collaboratively by JB and AGD to ensure interpretation of the Stories reflected Bunurong perspectives and the intended meaning and significance of the Knowledge shared.

Once the themes had been identified, a review of the scientific literature was conducted to explore how the ecological concepts interpreted from the Stories related to WS understandings of marine mammal ecology in Victoria. This process examined areas of convergence between Bunurong Knowledge and WS, as well as where the Stories identified ecological relationships, behavioural displays, habitat regions or historical perspectives that were absent from or underrepresented in the scientific literature. These areas of convergence and divergence between knowledge systems were then used to identify potential future research directions and conservation considerations emerging from the Bunurong Stories. The objective of this process was not to validate Bunurong Knowledge using WS, but rather to explore areas where the knowledge systems converged, complemented one another or generated new ecological questions.

### 2.5 Walagunh Baanhbula (Mixing Two Waters)

The initial methodological framework proposed by JB for this study centred on the weaving of IEK and WS knowledge systems. This concept describes the bringing together of multiple knowledge systems to facilitate equitable knowledge exchange and deeper learning (Henri et al. 2021). Similar approaches have been described as the ‘bridging’ or ‘braiding’ of knowledge systems (Alexander et al. 2019; Johnson et al. 2016) or ‘Two‐Eyed Seeing’ (Wright et al. 2019; Reid et al. 2021). Central to these frameworks is the recognition of Indigenous Knowledge as an equally valid and valuable way of knowing alongside WS (Henri et al. 2021; Johnson et al. 2016). However, during the development of this manuscript, AGD expressed a disconnect with the term ‘weaving’ and the concept of ‘interweaving of Indigenous Knowledge and WS’ in the context of Warrinh Biik and marine mammals. Instead, AGD proposed the concept of ‘Walagunh Baanhbula’—‘mixing two waters’—to describe the coming together of Indigenous and scientific knowledge systems.

Grounded in a freshwater/saltwater concept, Walagunh Baanhbula refers to places where waters meet and mix, creating areas of abundance and productivity. In this study, the concept represents more than just the coexistence of separate knowledge systems; it reflects their integration and mutual contribution to a more informed understanding of marine mammals and Sea Country. Rather than separate strands being woven together, Walagunh Baanhbula embodies the complete amalgamation of ecological knowledge, where distinct ways of knowledge meet, meld and strengthen one another.

This is a similar concept to the ganma metaphor, which originates from the Yolŋu Peoples from northeast Arnhem Land, Australia. Ganma can have two meanings; it can describe an area within a mangrove where saltwater comes in to meet freshwater. It also represents a process of knowledge production, where the saltwater and freshwater can come together and mix without losing their distinctiveness, where they can exist together in mutual respect (Muller 2014).

### 2.6 Bunurong Representation

These Stories have been written and shared by AGD and reflect her Knowledge and connection to Bunurong Sea Country. While the artwork created by JH reflects her interpretation of AGD's Stories and her own connection to Sea Country. The contents of these Stories were shared with AGD's permission for dissemination to the broader community; however, a greater depth of Knowledge also remains within the Bunurong community. The Stories, artwork and the manuscript have been shared with BLCAC for review and approval and have received support for dissemination.

We acknowledge that Indigenous Knowledge systems are diverse and can vary across families, lineages, genders, places and cultural, Totemic, spiritual and Custodial responsibilities. The Stories shared here represent the narrative and perspectives of AGD, **Mundigurrk nhilam** (Grandmother cranky), and while supported by BLCAC, they do not seek to represent all Bunurong, Kulin Nation or broader Indigenous perspectives.

### 2.7 Use of Language (Non‐English)

The majority of the language and names used throughout this study (Table 1) were shared by AGD and further documented by the Ngawak Ngul Language Program (https://bunurong‐language.org/). We acknowledge that variations in spelling and usage exist across Aboriginal Australian languages and communities. These variations reflect the longstanding oral transmission of language, as well as the ongoing process of revival and reconstruction.

**TABLE 1:** Glossary of traditional names/words, including Bunwurrung terms used throughout this work, along with other traditional words referenced in the text.

Word	English meaning	Nation
**Bagurrk‐nganhin**	Our women	Bunurong peoples of the Kulin Nation
**Barranan**	Dolphin	Bunurong peoples of the Kulin Nation
**Barrananbulayt**	Two dolphin	Bunurong peoples of the Kulin Nation
**Burrunan**	Dolphin	Boonwurrung, Woiwurrung, and Taungurung Languages of the Kulin Nation
**Baytayil**	Whale	Bunurong peoples of the Kulin Nation
**Baytayil Barndin**	Mother Whale	Bunurong peoples of the Kulin Nation
**Beowas**	Orca	Thaua people of the Yuin Nation
**Biik**	Country	Bunurong peoples of the Kulin Nation
**Bunwurrung**	Language of the Bunurong peoples	Bunurong peoples of the Kulin Nation
**Djudju**	Baby Whale	Bunurong peoples of the Kulin Nation
**Djuwap**	French Island	Bunurong peoples of the Kulin Nation
**Ganma**	Represents a process of knowledge production, where the saltwater and freshwater can come together and mix without losing their distinctiveness	Yolŋu People of the Miwatj region (northeast Arnhem Land)
**Iqalukjuaq**	Greenland shark	Inuit community in Pangnirtung in southern Baffin Island
**Karralk**	The place that waits in the west for those who leave us in death	Bunurong peoples of the Kulin Nation
**Kuyingkuying**	When spring is turning to summer	Bunurong peoples of the Kulin Nation
**Kurrman**	Seal	Bunurong peoples of the Kulin Nation
**Kurrmangurrk**	Seal woman	Bunurong peoples of the Kulin Nation
**Mātauranga Māori**	Māori Knowledge	Māori people of Aotearoa
**Mankarr**	Greater bilby	Martu people of the Martu Native Title Determination Area in Western Australia
**Milawul**	Phillip Island	Bunurong peoples of the Kulin Nation
**Mirngmum**	Seal (direct translation – eye bum)	Bunurong peoples of the Kulin Nation
**Miyapunu**	Marine turtle	Yolŋu People of the Miwatj region (northeast Arnhem Land)
**Mundigurrk**	Grandmother	Bunurong peoples of the Kulin Nation
**Mundigurrk nhilam**	Grandmother cranky	Bunurong peoples of the Kulin Nation
**Naarm**	Port Phillip Bay	Bunurong peoples of the Kulin Nation
**Ngamadjiyt**	White person	Bunurong peoples of the Kulin Nation
**Pulan**	Amity Point	Nunukul people of the Quandamooka people
**Pūrākau**	Legends	Patuharakeke (a composite hapū from most of the iwi groups in northern Aotearoa)
**Te Ākau**	Bream Bay	Patuharakeke (a composite hapū from most of the iwi groups in northern Aotearoa)
**Tohorā**	Southern right whale	Patuharakeke (a composite hapū from most of the iwi groups in northern Aotearoa)
**Turembulerrer**	Twofold Bay	Thaua people of the Yuin Nation
**Walagunh**	Mixes/mixing	Bunurong peoples of the Kulin Nation
**Walagiyt**	Mix	Bunurong peoples of the Kulin Nation
**Walagunh** **Baanhbula**	Two waters mixing	Bunurong peoples of the Kulin Nation
**Wamum**	Wilsons Promontory	Bunurong peoples of the Kulin Nation
**Warrinh**	Sea/ocean	Bunurong peoples of the Kulin Nation
**Warrinhgurrk**	Sea Women	Bunurong peoples of the Kulin Nation
**Warrinh Biik**	Sea Country	Bunurong peoples of the Kulin Nation
**Wirribi yaluk**	Werribee	Bunurong peoples of the Kulin Nation
**Wurnmarrinh**	Western Port Bay	Bunurong peoples of the Kulin Nation
**Yulendji Bunwurrung**	Bunurong Knowledge	Bunurong peoples of the Kulin Nation

### 2.8 Ethical Approval, Informed Consent and Indigenous Cultural and Intellectual Property Agreement

The RMIT University Human Ethics Committee (Approval number: 27507) approved this project in March 2025. This ethics application was supported by BLCAC, with a letter of support provided by the Corporation for the ethics application. A letter of support was also provided by BLCAC for funding applications.

Before the commencement of interviews, AGD signed a Participant Information Sheet and Consent From (PISCF), and before the submission of this manuscript, an Indigenous Knowledge Sharing and Authorship Agreement was signed to ensure that all dissemination of Knowledge and Stories was approved by AGD and credited to AGD.

An Indigenous Cultural and Intellectual Property (ICIP) Agreement was signed by AGD and JB to ensure that AGD remains the rightful Custodian of her ICIP. As part of the broader project, the artwork by JH was created to visualise AGD's Stories and enhance the Storytelling nature of this study. JB sought permission for these artworks to be included in this manuscript, and consent was granted.

## 3 Results

### 3.1 Yulendji Bunwurrung (Bunurong Knowledge); Stories Written by Aunty Gail Dawson and Artwork by Jayde Halls

#### 3.1.1 Bunwurrung Women Are From the Sea, of the Sea

The first two Bunwurrung women were created beyond a dancing curtain of kelp, from mud, coral and sand, and given custodianship over Sea Country and all that belongs to Warrinh, the ocean and the freshwater of Biik. Bunwurrung women hold the Lore of Warrinh, maintaining the balance of sea movements, sea creatures, plants, rocks, sand and coral and all being and non‐being below the water. Bunwurrung women follow their ancestors' ways, making sure that there is always food on the shores and under the sea. This knowledge is held by Bunwurrung women in their stories, ceremony, songs and dances.

Bunwurrung women are from the sea and can stay underwater for a long time. They know the creatures that swim beneath the waves, holding their breath until they surface to take a breath before diving again. Whales, dolphins and seals are sea‐women. They hold their babies safely within their bodies, then keep them close until they gather strength and knowledge before releasing them to an often‐dangerous world. All sea‐women keep their babies close, suckling them and teaching them to find their own food. Our sea‐family mammals swim with other females and the Mundigurrk—grandmother, literally the ‘embrace/hold women’, lead the family and fight to keep them safe.

Sealers and whalers saw that Bunwurrung women knew the whale's language, basked on the rocks with seals and called to dolphins for fish. These uncivilised men saw nothing but a resource to be taken for their enrichment. The brutal story of cruel enslavement and destruction of our sea‐family still haunts us as it haunts Mother Whale, Mirngmum and Barranan. We can't speak of this story without our bodies crying. We watch and wait for our Warrinhgurrk—our sea‐women and their families—to return to Naarm, growing in number and strength like the Bunwurrung people.

**FIGURE 2:**
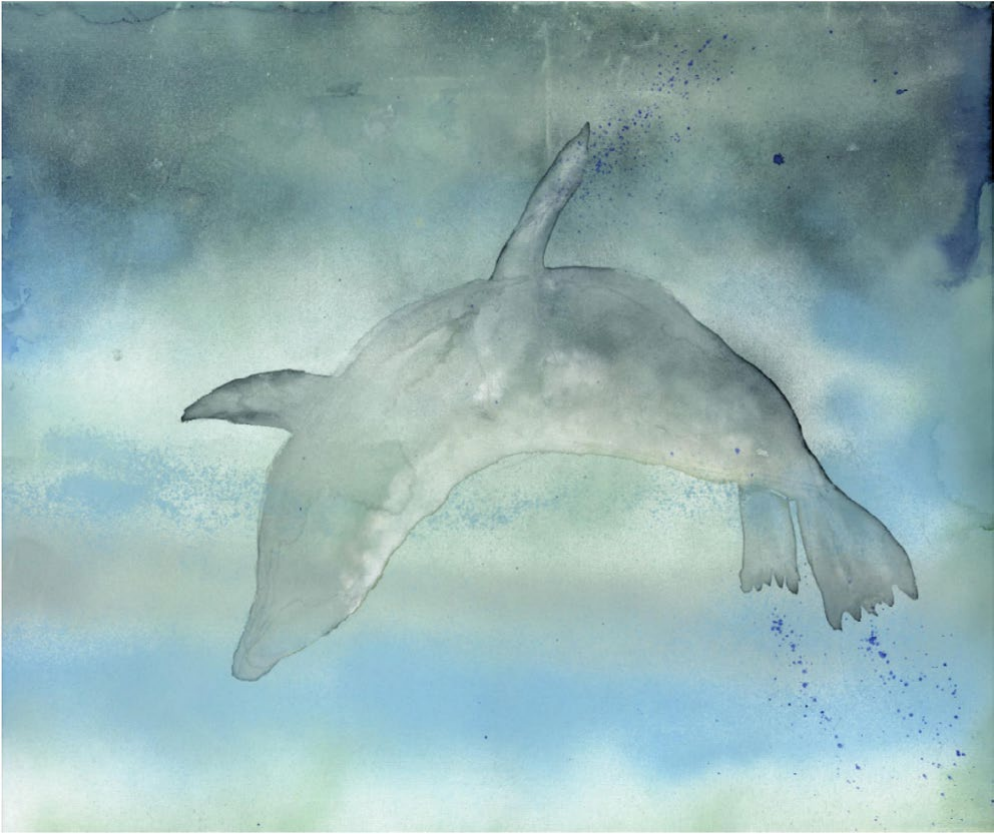
Kurrman (seal), artwork created by Jayde Halls.

Artwork description: Kurrman, sometimes called Mirngmum for her special skills, plays in the water after lying on the warm rocks with her clan and all the new pups (Figure 2). She is checking the water and listening for the sounds of food, her kin and danger. She knows Mundigurrk (grandmother) is watching over the babies.

#### 3.1.2 Barndin Baytayil Returns to Bunwurrung Bagurrk‐Nganhin (Mother Whale Returns to Bunwurrung Women)

Wurnmarrinh/Westernport is one place where Bunwurrung women and young children greet the whales resting on their long journey to warmer waters. Karralk is the place that waits in the west for those who leave us in death, riding with Mother Whale on the rays of the setting sun before all is darkness. When Mother Whale returns to our Sea Country with her calves, the women gather for ceremonies and watch carefully for the whales that carried our people to Karralk. We look for the shape, the way that whale moves, the way it looks and behaves.

When the whales return to Naarm, we tell the story of Djudju the baby whale and the creation of Wurnmarrinh, called Westernport in Ngamadjiyt language. The story is a cautionary tale for children and a way of knowing and finding our way on Country. As always, Mother Whale and Djudju swam back to Naarm to gather strength for the long journey to the cold south, sang with the Bunwurrung women gathered for ceremony and shared stories of old times. Djudju frolicked near the pod as his mother had taught him but heard the shouts and laughter of children playing on the shore. Before the grandmothers and mothers could block him, he launched his fat little body onto the land and lay gasping, unable to move. Mother Whale swam out to the deeper water, then turned and rushed to the shore, diving onto the land and threshing round and round Djudju's struggling body, opening a water path for her calf to swim back to the pod. The place where Djudju struggled is called Djuwap by the Bunwurrung, French Island in English.

Mother Whale holds the spirits of Bunwurrung children, and sometimes when whales hear the children's voices, they swim onto the land, trying to find their old camp from long ago before they went to Sea Country. When the whale beaches, the people gather for the crying ceremony to honour the creature who works with them to keep ocean harmony. Those whose moiety and family line hold authorisation to eat whale flesh take the whale spirit back into their body, keeping the whale's spirit alive in a way that honours the Deeptime inter‐connectedness of humans and the world created by the Ancestors.

Following the monstrous slaughter by sealers and whalers, whales stopped coming for a long time, throwing the balance of Biik and creation into chaos. Now the whales, seals and dolphins are coming back, like the Bunwurrung people stolen from their Biik, to restore the interconnectedness and balance that keeps the world strong and safe.

**FIGURE 3:**
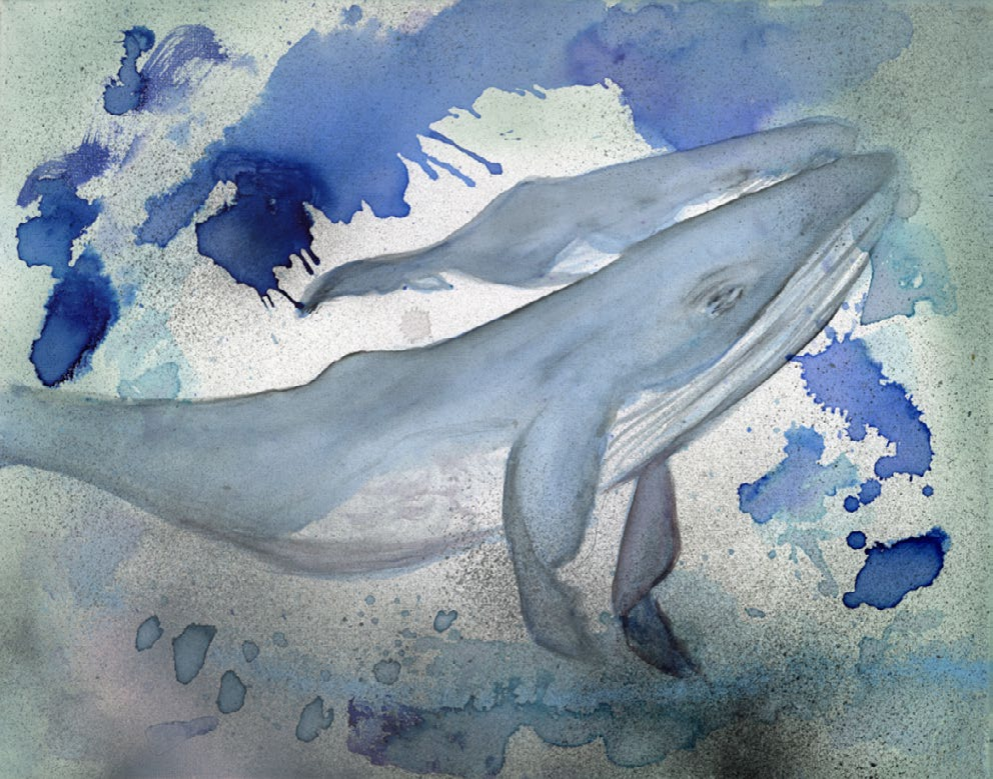
Barndin Baytayil (Mother whale) and Djudju (baby whale), artwork created by Jayde Halls.

Artwork description: Barndin Baytayil and Djudju swim close together, close like Bunurong mothers and babies (Figure 3). She feeds her baby milk, and Djudju learns how to be a whale by watching and listening to her voice during their long journey back to Country.

#### 3.1.3 Barranan Talks With Baytayil

Dolphin talks to Whale, and they understand each other because they are companion animals. Barranan is related to Mother Whale—sometimes her husband, sometimes her brother. Barranan escorts the returning pod back into Naarm, and they share stories about Baytayil's long journey to the warm northwestern waters to mate and strengthen the newborn calves before returning to Naarm. They share stories of loss, attacks by predators, strandings, the tangling nets and the ships that cut through the water without listening or watching. There are also stories of the glorious glide through the kelp forests and the reef that survives the now bitter water they swim through.

**FIGURE 4:**
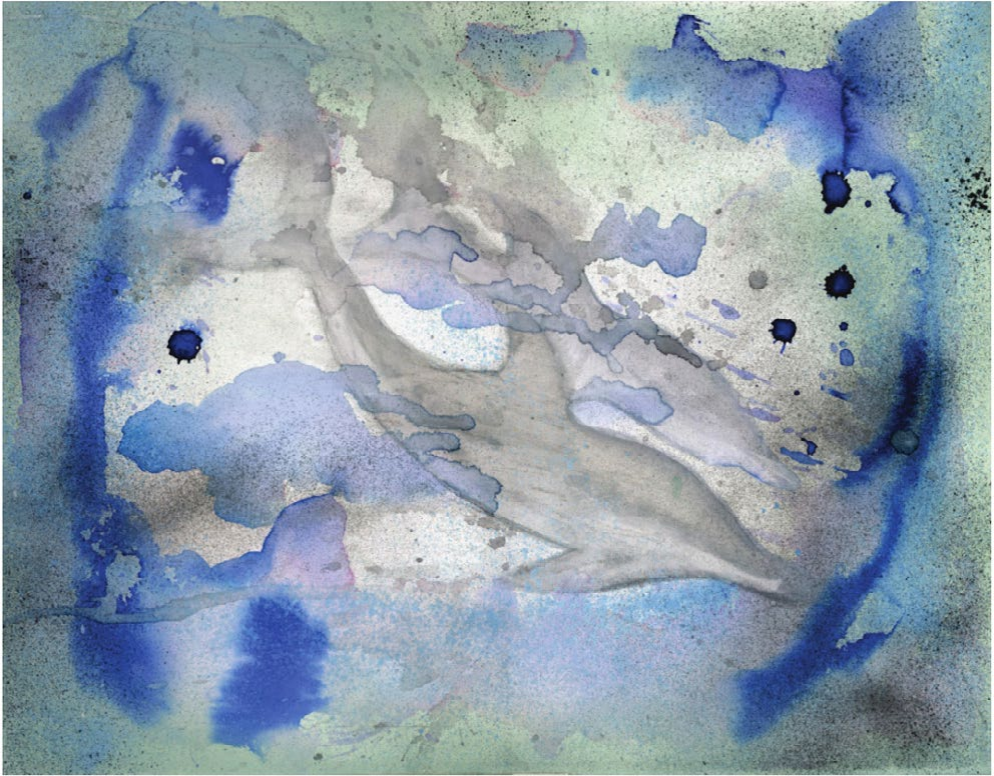
Barranan (dolphin), artwork created by Jayde Halls.

Artwork description: Barrananbulayt (two dolphins) hear the women singing and clapping the water (Figure 4). They look at each other and grin, call loudly for their pod, who come from all around. They round up the fish and herd them towards the shallow water into the women's nets.

#### 3.1.4 Kurrman Basks on the Rocks and Sings With the Women

Bunwurrung women stay safe in the ocean with the protection and knowledge given by the kelp‐woman, an ancestral being of the sea. She is known by many names and has many forms, and one of her names is Kurrmangurrk—sealwoman—because she came from the seal in another time. She still swims with seals, dolphins, whales and Bunwurrung women, showing them the stories and secrets of Warrinh.

Some women lie still and quiet with the seals or lie with them in the water with their arms raised, until they recognise her as one of their own. There is agreement that at times the women will take a seal and share the meat with those who have the right to eat that meat. Eating the meat of kurrman keeps the ancient bonds of connection between those that share Warrinh and work together to keep Sea Country in balance. We have strict rules about which seal can be taken for meat, and only in certain seasons.

Seals can see with other parts of their body, not just their big eyes, seeing things far away that we can't, which gives them special knowledge of the underwater and shoreline. Like Bunwurrung women, whales and dolphin, they live above and below the sea. We see their pups at the same time as Bunwurrung women are having their birthing ceremonies, as summer begins and there is plenty of food for babies. The pups have to grow up quickly because soon there will be more pups for mother to feed and protect. Mundigurrk stories about Kurrmangurrk recount how female seals also use sea‐woman knowledge to manage the time of birth, and they keep babies in their body for the same time as Bunwurrung women. Their babies are born when Bunwurrung women gather on the gentle waters of Naarm to welcome babies. Mother seal stays close to her pup, sharing care with grandmother seal and the other females in her family group. She keeps feeding the pup on her milk until next Kuyingkuying—September, when spring is turning to summer.

Before the sealers and colonists came and took too many seals, there were seals all over Naarm and at Wamum on the southern tip of Bunwurrung Country. Now they are coming back, and the people wait until kurrman covers the rocks and Baytayil and Barranan swim in the two bays. We keep telling our sea‐women stories when we are all sitting down doing gentle work, or tracing the stories in the night sky, or walking on a sea‐woman songline. We know that no matter how we are hunted and killed, by telling our stories, we keep safe the knowledge that keeps Country and all it holds safe and balanced.

**FIGURE 5:**
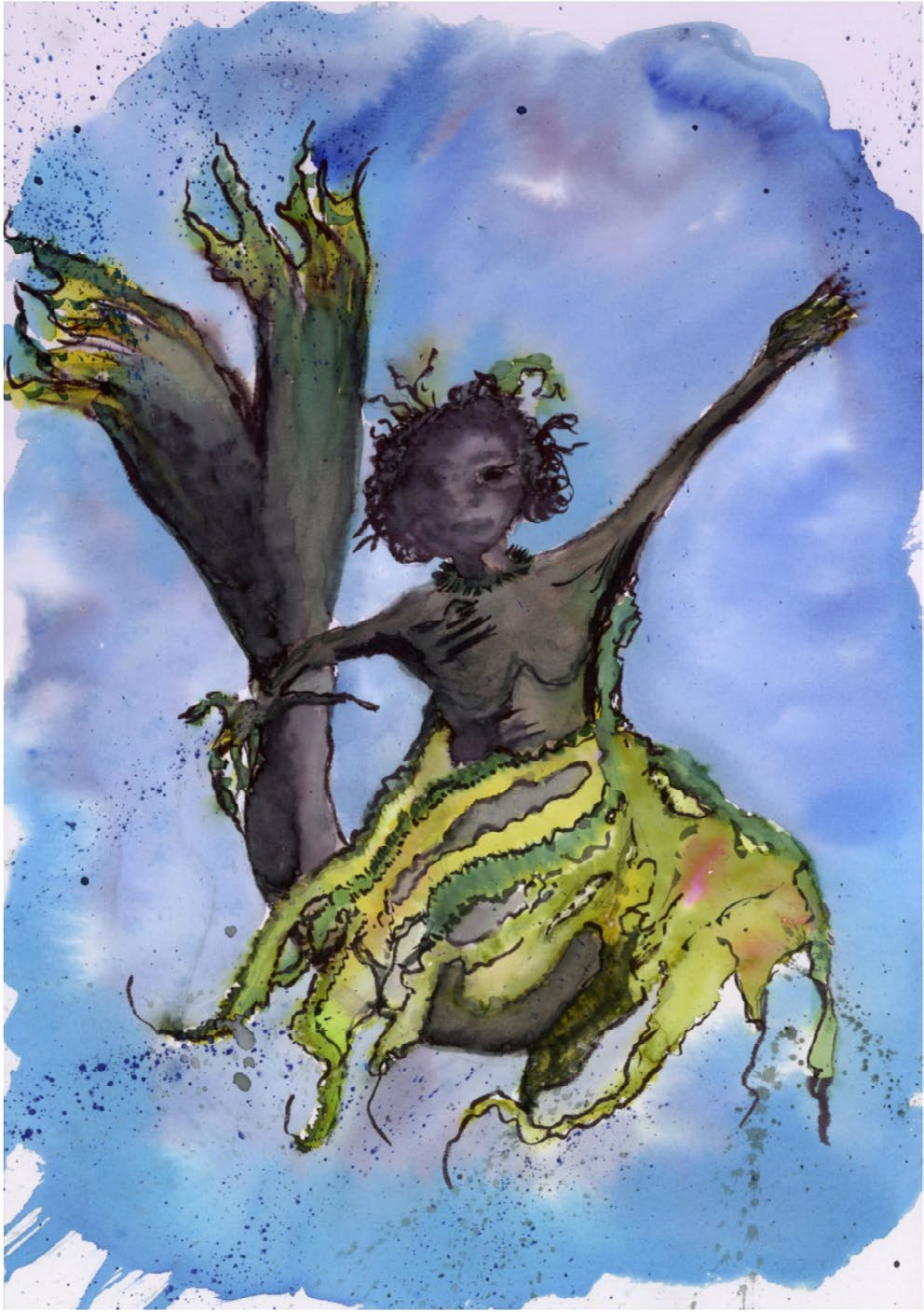
Kurrmangurrk (sealwoman) artwork created by Jayde Halls.

Artwork description: Kurrmangurrk is a kelpwoman who came from a seal in another time (Figure 5). Kurrmangurrk has many names because she takes many shapes as she protects Bunurong women hunting in the sea and reminds them of the ways to keep Warrinh healthy and strong.

#### 3.1.5 Bunurong/Boon Wurrung—Barranan Protects Bunwurrung Children and Brings Fish

Bunwurrung women dive for shellfish and collect kelp to make baskets, and under the sea, they talk with Barranan the dolphin, sharing stories and knowledge. Women and children swim with Barranan, playing games and singing to each other, and if a child struggles in the water, Barranan holds the little one on his head and pushes the child to the safety of the shore. When the signs of Sky Country and Biik tell them to leave their inland estates, the clan returns to the gentle waters of the bay. Women who hold the dolphin story stand in the water and sing to Barranan, and the Barranan pod laughs and sings as they round up the fish and herd them into the shallow waters to the women's string nets.

We have been singing and playing with dolphins for over 5000 generations, learning how to work with them, listening and passing dolphin knowledge down the women's family lines. Our grandmothers' dolphin stories remind us of the way all sea‐women behave, the females staying together, sharing the care and protection of children until they are ready to leave the women's camp. Female dolphins stay together, protecting each other and the calves from predators and teaching them to hunt. Like the grey‐haired Bunwurrung Mundigurrk who moves slowly but fights fiercely to keep her family safe, Grandmother Dolphin has lived a long time, so she shares her knowledge with the young women and children. Male dolphins stick together or live alone, but when they arrive in cold season, they make us laugh when they show off to the females with crazy dancing and singing. Bunwurrung women stay out of the water when they see the gangs of males working together to get close to the female because they can be very rough.

Dolphin Law about husband and wife is like the rules that tell us not to marry ‘wrong side’, so the males might swim south when the weather gets warmer, then return to Naarm when the southern ocean gets too cold. The females are like Bunwurrung women, having their babies in spring and summer when there is plenty of food, giving them milk until they are old enough to feed themselves.

**FIGURE 6:**
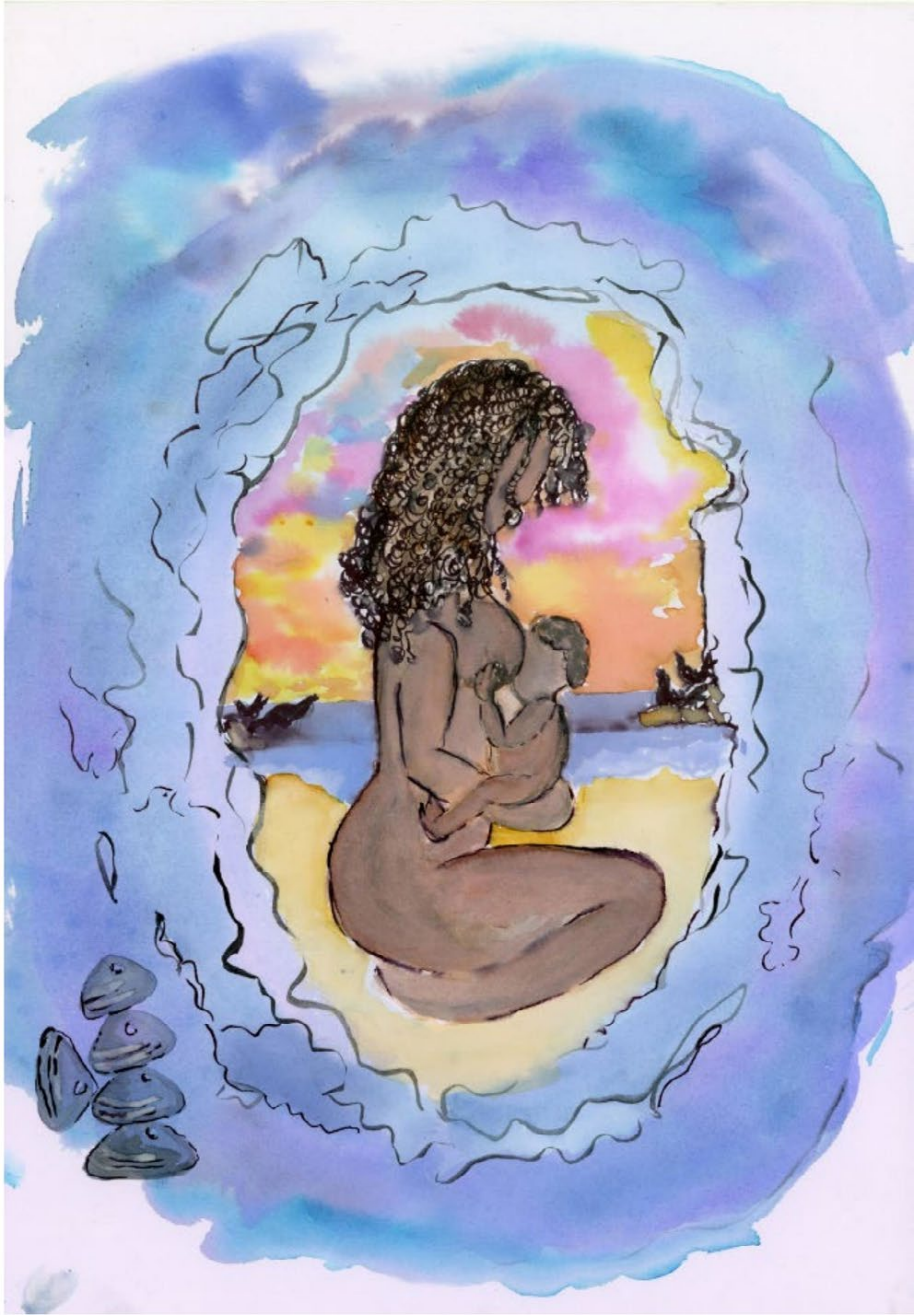
Bunwurrung woman and child, artwork created by Jayde Halls.

Artwork description: The Bunwurrung woman and her child sit with their seal family (Figure 6). The kurrman hears the voice of her new baby and smells her scent on the breeze. She is teaching bubup about her seal family, and she is giving the kurrman the name, voice and scent of her baby so they will always know she is family.

### 3.2 Major Themes Interpreted From Yulendji Bunwurrung

**FIGURE 7:**
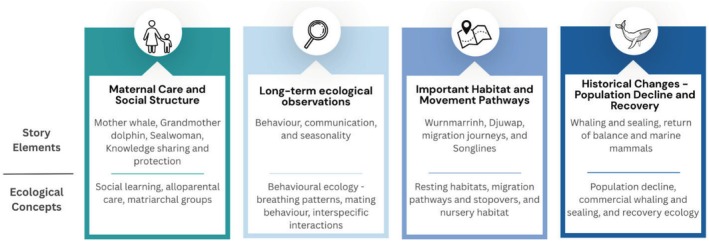
Themes interpreted from thematic analysis of Bunurong Stories.

Thematic analysis of the five Bunurong Stories identified four key themes that provide insights into marine mammal ecology and the relationships between marine mammals, Bunwurrung women and Sea Country (Figure 7). The themes described maternal care and social structure, long‐term ecological observations, important habitats and movement pathways and historical changes associated with population decline and recovery. Together, these themes demonstrate how ecological knowledge is embedded within Story through observations of marine mammal behaviour, habitat use, seasonal movements and changes in response to anthropogenic pressures. The themes also illustrate the interconnected nature of ecological, cultural, spiritual and social knowledge within Bunurong understandings of Sea Country.

#### 3.2.1 Marine Mammal Maternal Care and Social Structure

Stories describing **Baytayil** (whale), **Barranan** (dolphin) and **Kurrman** (seal) emphasise maternal care, intergenerational knowledge sharing and female leadership. Across all species, mothers were described as maintaining close associations with offspring, providing prolonged maternal care for their young, protection, being teachers and sharing their knowledge. Grandmothers were also identified as important leaders and knowledge holders within the Bunurong community and marine mammal social groups.

Examples:

Like the grey‐haired Bunwurrung Mundigurrk who moves slowly but fights fiercely to keep her family safe, Grandmother Dolphin has lived a long time so she shares her knowledge with the young women and children.

Sealers and whalers saw that Bunwurrung women knew the whale's language, basked on the rocks with seals, and called to dolphins for fish.

All sea‐women keep their babies close, suckling them and teaching them to find their own food. Our sea‐family mammals swim with other females and the Mundigurrk ‐ grandmother, literally the ‘embrace/hold women’, lead the family and fight to keep them safe.

#### 3.2.2 Long‐Term Ecological Observations

The Stories contained observations relating to marine mammal behaviour, including breathing patterns, communication, social structure, mating behaviours, maternal care, migration patterns, habitat use and seasonal timings. The observations were not just embedded in Story, but also in ceremony and cultural practice.

Examples:

Bunwurrung women are from the sea and can stay underwater for a long time. They know the creatures that swim beneath the waves, holding their breath until they surface to take a breath before diving again.

Dolphin talks to Whale, and they understand each other because they are companion animals.

Bunwurrung women stay out of the water when they see the gangs of males working together to get close to the female because they can be very rough.

#### 3.2.3 Important Marine Mammal Habitat and Movement Pathways

Within the Stories, multiple locations in Bunurong Country were specified as important areas for marine mammals. **Wurnmarrinh** (Western port Bay) was described as a location where whales may rest during their migration journey and where mothers and calves gather together. **Djuwap** (French Island) is identified as a place important for whales and human‐whale relationships. Seasonal movement patterns and journeys through Sea Country were also described.

Examples:

Wurnmarrinh/Westernport is one place where Bunwurrung women and young children greet the whales resting on their long journey to warmer waters.

Barranan escorts the returning pod back into Naarm, and they share stories about Baytayil's long journey to the warm northwestern waters to mate and strengthen the newborn calves before returning to Naarm.

#### 3.2.4 Historical Changes—Population Decline and Recovery

The impacts of colonisation and the commercial sealing and whaling industry are referenced throughout the Stories, and periods where whales, seals and dolphins became less frequent in Bunurong waters. These accounts were accompanied by descriptions of contemporary returns of marine mammals and the return to balance for Sea Country.

Examples:

Following the monstrous slaughter by sealers and whalers, whales stopped coming for a long time, throwing the balance of Biik and creation into chaos. Now the whales, seals and dolphins are coming back, like the Bunwurrung people stolen from their Biik, to restore the interconnectedness and balance that keeps the world strong and safe.

We watch and wait for our Warrinhgurrk ‐ our sea‐women and their families ‐ to return to Naarm, growing in number and strength like the Bunwurrung people.

## 4 Discussion

### 4.1 Marine Mammal Maternal Care, Social Structure and Ecological Observations in Bunurong Stories

Throughout the Stories, there is a strong emphasis on **Baytayil Barndin** (Mother whale) and the protection of **Djudju** (Baby whale), which aligns with observations of strong maternal investment in both humpback whales ( *Megaptera novaeangliae* ) and southern right whales ( *Eubalaena australis* ). It has been documented across the majority of cetacean species that mothers and calves remain together for extended periods of time following birth, with mothers investing heavily in each of their offspring (Foroughirad et al. 2026). Toothed and baleen whales exhibit differing maternal strategies, with toothed whales typically focusing on slow growth, prolonged maternal care and social learning, whereas baleen whales prioritise rapid milk transfer and accelerated calf growth (Foroughirad et al. 2026). Both the humpback whale and southern right whale have displayed strong social cohesion when defending their young. Humpback whales have been observed mobbing killer whales and responding to attacks through active physical defence behaviours, including thrashing, rolling, slashing tail flukes and head shaking (Pitman et al. 2017; Ford and Reeves 2008). In contrast, the slower but more robust southern right whale has been seen to form a ‘rosette’ around vulnerable calves to protect against predators (Ford and Reeves 2008; Foroughirad et al. 2026). These species exhibit extensive periods of calf dependency, with mothers providing nutritional support and physical protection from predators during early life stages.

Instances where menopause is known (or probable) occur within species that all have matrilineal social systems (McAuliffe and Whitehead 2005). Post‐reproductive cetacean females are thought to provide benefits for their social group, either directly or indirectly via sharing the information they possess. Intergenerational help has been identified as one of the key benefits of living long in species with menopause (Ellis et al. 2024). Older females or ‘grandmothers’ take on a unique leadership role and can help by babysitting their grand‐offspring, sharing food with their relatives or using their ecological knowledge to help find resources (Ellis et al. 2024; Foroughirad et al. 2026). Similar to how the grey‐haired **Bunwurrung Mundigurrk** (Bunurong grandmother) looks after her family, shares her knowledge and protects her kin, these Stories mirror the emerging evidence that post‐reproductive females can provide significant ecological and social benefits within cetacean societies.

The Burrunan dolphin (*Tursiops australis*) also exhibits highly complex social structures, with sub‐groups typically characterised by same‐sex associations. Females commonly form nursery pods, with female offspring remaining with their mothers and female relatives into adulthood, whereas males usually seek social interactions away from their mothers, often establishing cooperative pairs (dyads) or trios (triads) (Crittenden et al. 2025). Dolphin male alliances can engage in sexual coercion, evidenced by aggressive behaviours such as raking (Scott et al. 2005), disruption of regular female ranging and behaviour (Wallen et al. 2016) and direct aggression towards females (Connor et al. 1996), a behaviour observed within Bunurong Stories. Furthermore, these matrilineal structures are characteristic of several odontocete species (Aubin et al. 2026) in which older females play important social and ecological roles within family groups. Although menopause has not yet been confirmed in the Burrunan dolphin, the species belongs to the family Delphinidae and is therefore closely related to several odontocete species in which menopause has been documented (Karniski et al. 2018), including false killer whales ( *Pseudorca crassidens* ), killer whales/orca ( *Orcinus orca* ) and short‐finned pilot whales ( *Globicephala macrorhynchus* ), belugas ( *Delphinapterus leucas* ) and narwhals ( *Monodon monoceros* ) (Aubin et al. 2026; Foote 2008; Photopoulou et al. 2017). Further investigation into the social roles of older female Burrunan dolphins may therefore provide valuable insights into the evolution and ecological significance of post‐reproductive life histories within the species.

Interspecific interactions between cetacean species have been reported on a number of occasions (Koper and Plön 2016) and can vary greatly in their function, including predatory, playful, sexual and caregiving interactions (Deakos et al. 2010). From a global review of interactions between baleen whales and dolphins, Meynecke and Crawley (2025) found that a quarter of interactions were positive and that these interactions may be more common and complex than originally thought. In the positive interactions, the humpbacks were seen to be partaking in playful behaviours including rolling from side to side, presenting their bellies and other activities that are typically associated with courtship behaviour or socialising. The interspecific engagement did not just occur at the sea surface but was also reported to occur along the ocean floor as well. The Stories describe a companionship between Baytayil and Barranan that extends beyond simple co‐occurrence. While interspecific interactions between dolphins and baleen whales have been documented globally (Meynecke and Crawley 2025), little is known about the frequency or ecological significance of these interactions within Victorian waters. Observations described in Bunurong Story, together with contemporary sightings of Humpback whales and Burrunan dolphins interacting in **Naarm** (Port Phillip Bay) and Wurnmarrinh (K. Robb, personal communication), suggest that these interactions may occur within Victorian waters. However, these observations have not yet been examined in the scientific literature, highlighting a potential area for future research.

### 4.2 Knowledge of Habitat Use in Bunurong Stories

#### 4.2.1 Baytayil: Whale Habitat and Migration Pathways

The Bunurong Stories describe Baytayil returning to Naarm from northwestern waters, a movement pattern that differs from the contemporary understanding of humpback whale migration along the Victorian coastline, which is typically associated with the eastern Australian breeding population travelling between Antarctic feeding grounds and breeding areas in Queensland (McPhee‐Frew et al. 2025). While most humpback whales observed within Victorian waters are presumed to belong to the eastern Australian stock, interchange between the eastern and western Australian sub‐stock has been seen (Seyboth et al. 2023; Gales et al. 2009). Consequently, reference to a return from northwestern waters within the Stories may reflect ecological observation or historical movement patterns that are not fully represented within current datasets. Further investigation into the origins and movement pathways of whales travelling through Victorian waters may help explore the convergence and divergence between Bunurong Knowledge and current scientific understandings.

While this interpretation is discussed in relation to humpback whales due to their well‐documented migration, the Stories may also refer to southern right whales. Southern right whales are frequently associated with mother‐calf pairs in Victorian waters and are commonly observed within recognised nursery habitats near Portland (O'Shannessy et al. 2025). Interestingly, biologically important areas for southern right whale reproduction have also been identified in Exmouth, Western Australia, suggesting that northwestern Australian waters may hold reproductive significance for multiple Baytayil species. Whether the reference to northwestern waters in the Story reflects historical movements of southern right whales, humpback whales or a broader Bunurong understanding of whale movement remains unclear and warrants further investigation.

In the present day, Wurnmarrinh supports a diversity of marine mammals; however, studies examining the use of the bay by Baytayil species remain extremely limited. Based on sighting records from the Global Biodiversity Information Facility (GBIF) since 1948, 425 cetacean sightings have been logged in Wurnmarrinh. This includes a range of species including the humpback whale, southern right whale, dwarf minke whale ( *Balaenoptera acutorostrata* ), fin whale ( *B. physalus* ), pygmy right whale ( *Caperea marginata* ), pygmy sperm whale ( *Kogia breviceps* ), sperm whale ( *Physeter macrocephalus* ), Grey's beaked whale ( *Mesoplodon grayi* ), long‐finned pilot whale ( *G. melas* ), killer whale/orca, bottlenose dolphin ( *T. truncatus* ), Burrunan dolphin, and the common dolphin ( *Delphinus delphis* ). Collectively, this showcases the habitat suitability of this region for cetaceans. Despite this, Wurnmarrinh is now home to frequent commercial shipping activity, with the Port of Hastings handling crude oil, LPG, steel and fuel (Kirkman 2013). Commercial shipping poses a risk to marine mammals through exposure to chronic underwater shipping noise (Redfern et al. 2017; Blair et al. 2016) and the increased likelihood of ship strikes (Mayaud et al. 2022), potentially impeding the return of marine mammal populations to the region. Baytayil sightings within Wurnmarrinh have become increasingly prominent, with humpback whales recorded annually since 2014. Notably, 2025 recorded the highest number of sightings, with 20 humpback whale observations. The importance of Wurnmarrinh for Baytayil, specifically for Baytayil Barndin, Djudju and Bunurong ceremony, is repeatedly mentioned throughout the Stories. Within the Stories, the Bay functions as a place of ceremony, knowledge transmission and ongoing relationships between Bunurong peoples and Baytayil. This highlights how culturally significant habitats may also represent ecologically important locations that may not have yet been prioritised by contemporary research or management frameworks.

#### 4.2.2 Barranan: Dolphin Habitat Within Naarm and Connection With Bunwurrung Women

The Bunurong Stories describe Barranan males travelling south during warmer months and returning to Naarm when the ocean temperatures cool, while females remain within the Bay to give birth and raise calves. These observations suggest sex‐specific differences in habitat use and movement patterns, reflecting seasonal shifts in the contraction and expansion of distribution ranges in response to breeding and/or birthing seasons (Clutton‐Brock 1997; Greenwood 1980; Sprogis et al. 2016). These shifts have been documented in bottlenose dolphin populations, where females tend to have a smaller home range (Gubbins 2002; Smith et al. 2013), while the males may have larger home ranges, especially during nonbreeding seasons where they may broaden their home range to optimise prey intake (Sprogis et al. 2016; Sprogis et al. 2018). Movement of the Burrunan dolphin beyond Naarm remains poorly understood. While direct observations and genetic analyses have demonstrated the occurrence of the species beyond Naarm, including identification of a Port Phillip Bay resident male in Portland, southwest Victoria (Charlton‐Robb et al. 2015), the extent to which resident individuals move beyond Naarm is unclear. Sex‐specific distributions and the presence of potential subgroups within the Port Phillip Bay resident population have been hypothesised (Beddoe et al. 2024), and stable isotope analyses suggest that individuals may also utilise offshore habitats, including Bass Strait, for foraging (Owen et al. 2011; Beddoe et al. 2024). Consequently, the Stories identifying movements through Bass Strait and beyond Port Phillip Bay represent an important area for future research.

Burrunan dolphins also occur south of Victoria in Tasmania, and a resident population is known to inhabit the Gippsland Lakes, east of Port Phillip Bay. Interestingly, population genetic analyses have found greater genetic similarity between the Gippsland Lakes population and Tasmanian populations than between either population and the Port Philip Bay population, suggesting limited contemporary gene flow between these regions (Charlton‐Robb et al. 2015). However, it is possible that geomorphological changes associated with the flooding of Bass Strait and the formation of Port Phillip Bay may have contributed to periods of population isolation (Holdgate et al. 2011), potentially contributing to the observed genetic dissimilarity and the formation of resident populations (Charlton et al. 2006).

The Stories also references reciprocal interactions between Barranan and Bunwurrung women, with the women singing to the dolphins as the dolphins kick up fish into the women's nets. These kinds of human‐dolphin interactions have long been documented across the world, including eastern Australia, Brazil, west Africa and the Mediterranean (Neil 2002). Cooperative foraging between humans and dolphins in southern Brazil (Cantor et al. 2023; Zappes et al. 2011) and in the Ayeyarwady river in Myanmar are the only known representations of mutualism still active today (Cram et al. 2022; Than Tun 2007). Fairholme (1857) documented ‘Porpoises’ (dolphins were commonly referred to as porpoises until the 1970s; despite being outside the normal range of any porpoise species) (Neil 2002) assisting the Quandamooka people fishing for mullet around **Pulan** (Amity Point) in the Moreton Bay region. Similar records document these activities occurring in Fraser Island, Stradbroke Island and along the northern New South Wales coast (Neil 2002). Further, there is documentation of cooperative hunting with **Beowas** (Orca) and the Thaua people of the Yuin Nation in **Turembulerrer** (Twofold Bay; Neil 2002; Reeves et al. 2023). The Beowas would assist in hunting baleen whales, typically humpback whales. Given the contemporary distribution of the Burrunan dolphin in Naarm and Wurnmarrinh, it is plausible that the Story refers to interactions between Bunurong women and resident Burrunan dolphin populations.

#### 4.2.3 Kurrman: Seal Habitat and Historical Areas of Importance

Australian fur seals ( *Arctocephalus pusillus doriferus* ) are known to range across south‐eastern Australia (McIntosh et al. 2022). On the outskirts of Wurnmarrinh is the largest breeding colony, known as Seal Rocks on **Milawul** (Phillip Island) (McIntosh et al. 2022), with another well‐known breeding colony near Wamum, known as Kanowna (Kirkwood et al. 2005). Australian fur seal births occur in early summer, typically from late October to late December (Warneke and Shaughnessy 1985), which often corresponds with peak food availability for adults and favourable temperatures for pups (Gibbens and Arnould 2009). The typical lactation period of the Australian fur seal is approximately 10 months; however, some mothers may continue to suckle their pup into a second or third year (Spence‐Bailey et al. 2007). This knowledge is reflected in Stories with Kurrman observed feeding their young until **Kuyingkuying** (when spring is turning to summer). Naarm is also home to a non‐breeding haul‐out site which is predominantly occupied by subadult and non‐territory‐holding male Australian fur seals at Chinamen's Hat (Speakman et al. 2020). Knowledge of Kurrman movement patterns and population size pre‐sealing remains limited, with abundance estimates largely inferred from seal skin export records (Kirkwood et al. 2005). Habitat predictability modelling by Bartes et al. (2024) indicates that Naarm and Wurnmarrinh currently offer a lower likelihood of hunting success for Australian fur seals compared to other regions of Bass Strait; however, these regions still provide some foraging potential. Adaptation of benthic foraging may also have occurred due to low marine productivity within Bass Strait (Spence‐Bailey et al. 2007), offering a potential explanation for the decreased habitat suitability in shallower embayments. However, further investigation into how Australian fur seals historically inhabited Naarm and Wurnmarrinh, and how these regions might support future conservation and population recovery, is encouraged.

It must also be noted that the discussion of marine mammal movement in Victoria is of current‐day (post‐colonial) records. Due to climatic changes and anthropogenic impacts, species that once inhabited the region may no longer be found. An example of this is evidence that the Dugong ( *Dugong dugon* ), a species which is currently restricted to the Indo‐Pacific region, once inhabited Naarm (Fitzgerald 2005). Therefore, while the above patterns may align within yulendji Bunwurrung, there is more to explore about the existence of these particular species historically. Furthermore, the southern hemisphere is seeing a poleward shift of marine species due to climate change (Hastings et al. 2020) which will likely see a continued shift in species movement patterns. Bunurong Stories identify Wurnmarrinh, Naarm, and associated Sea Country regions as culturally significant marine mammal habitats, highlighting locations and ecological relationships that remain underrepresented in contemporary marine mammal research and management.

### 4.3 Stories as Historical Ecological Records

The sealing and whaling industry played a significant role in the colonial history of Australia (Coutts 1984). It was sealers who found their ways to remote islands in both Australia and New Zealand, including Milawul in Wurnmarrinh. There are multiple historical accounts and documentation of how Aboriginal women, girls and boys were kidnapped and forcibly taken from their homes by sealers and whalers in the 1800s. They were then made to live with, work for and bear children for their captors (Edmonds 2014b). The survival of the sealers on islands in the Bass Strait and Kangaroo Island would not have been possible without Aboriginal women violently taken from the southern coast of Australia and Tasmania (Taylor 2000). These women knew how to live on the land, skilfully building shelters, catching and diving for fish and shellfish, finding water and stitching jackets from kangaroo skins, to name a few skills. While these atrocities committed by invaders, sealers and whalers are acknowledged, the ongoing Custodianship of Country by Indigenous peoples during this period, and the continuation of Knowledge through Stories, Songs, dances and Ceremony, are also recognised.

Both the humpback whale and southern right whale were heavily targeted by the commercial whaling industry. The southern right whale was the primary focus of whalers from the mid‐16th century through to the late 20th century, hunted for its blubber, which was rendered into oil; an estimated 58,000 individuals were killed in Australia and New Zealand (Stamation et al. 2020). The name ‘right whale’ derives directly from the whales being considered the ‘right whale to kill’ (Grundlehner et al. 2025). In contrast, the humpback whale population was depleted during modern commercial whaling between 1903 and 1973, with an estimated 216,000 individuals killed (Seyboth et al. 2023). Humpback whales in the southern hemisphere are thought to have recovered to nearly 80% of their pre‐whaling abundance (Seyboth et al. 2023). However, the recovery of the southern right whale has been considerably slower; current estimates suggest the western Australian southern right whale population has recovered to only 26% of its pre‐whaling abundance (Grundlehner et al. 2025). Beyond the current cetacean species that still frequently occur along the Victorian coastline, there were massive declines in most great whale species in the Southern Hemisphere due to unregulated commercial whaling. This includes the decline of blue whales ( *B. musculus* ), fin whales, sei whales ( *B. borealis* ) and sperm whales, which have not yet recovered and remain threatened to this day (Woinarski et al. 2015). Except for the sei whale, these species remain in the region according to the stranding record (Foord et al. 2019); however, their numbers are limited, and the species are listed as vulnerable (sperm whale) and endangered (blue whale and fin whale).

In the 1700s (prior to commercial sealing), it is estimated that there were 13–26 Australian fur seal breeding sites across Victoria and Tasmania (Pemberton and Gales 2004). From 1945 to 2000 there were nine breeding sites recognised (Pemberton and Gales 2004); however, now 26 breeding colonies are recognised in Victoria and Tasmania (McIntosh et al. 2022), with expansion primarily seen in Tasmania. Before the population was exploited during the commercial sealing era, it is estimated that the population was approximately 200,000, with 50,000 pups annually (Kirkwood et al. 2005). Currently, the Australian fur seal is one of the most geographically constrained fur seal species (McLean et al. 2018), with a generalised estimate for population size in 2017 being 89,5000 seals (McIntosh et al. 2022).

Bunurong Stories provide observations that pre‐date scientific monitoring and therefore may contribute to understanding historical marine mammal distributions and abundance. These Stories offer awareness into historical abundances and habitat use that predate colonial impacts, while also challenging assumptions about what is considered a healthy or recovering marine ecosystem.

### 4.4 Walagunh Baahnbula (Mixing Two Waters)

The thematic analysis and following literature review identified multiple areas where Bunurong ecological knowledge and contemporary marine mammal science converge. These areas of convergence reveal opportunities for collaborative research and may assist in identifying previously overlooked habitats, behaviours and conservation priorities.

It is important to recognise that, while this study explores the ecological observations contained within these Stories, they also convey cultural teachings, responsibilities and Lore. The Stories emphasise the importance of sharing Knowledge to maintain balance within Country and describe the enduring relationships between people, marine mammals and Sea Country. Teachings such as learning from Barranan, the sharing of secrets and Stories from **Kurrmangurrk** (sealwoman), and the creation of Wurrmarrinh when Djudju swam too close to the shore illustrate the cultural meaning and guidance that extend beyond ecological interpretation. These elements have no direct equivalent within WS, yet they represent a fundamental component of Indigenous Knowledge systems and form an integral part of the teachings conveyed through the Stories.

#### 4.4.1 Convergence Between Knowledge Systems

Several ecological observations contained within the Bunurong Stories align with contemporary understandings of marine mammal ecology. These areas of convergence include descriptions and knowledge of maternal care and prolonged dependency of young, female‐led social structures, social learning and sharing of Knowledge from Grandmother figures, seasonal movements and migrations and the historical impacts of commercial sealing and whaling. Together, these themes show substantial overlap between Bunurong ecological Knowledge and WS understandings of marine mammal behaviour and life history. However, the Stories also describe ecological relationships, behaviours and habitat associations that are poorly understood or underexplored within the WS community.

In addition to these broader ecological themes, Stories told by AGD also contain descriptions of marine mammal behaviours that correspond with contemporary observations. These include references to Bunwurrung women who ‘basked on the rocks with seals’, marine mammals ‘holding their breath until they surface to take a breath before diving again’, and seals occupying both terrestrial and marine environments as ‘they live above and below the sea’. These observations indicate a detailed understanding of marine mammal behaviour and ecology, suggesting that the Stories preserve ecological knowledge accumulated through generations of observing and interacting with marine mammals and Sea Country.

#### 4.4.2 Complementary and New Research Directions

While many of the observations described in the Stories closely align with WS knowledge, other aspects—including descriptions of interspecific relationships between Baytayil and Barranan, the significance of Warnmarrinh as a place of refuge for Baytayil, and reciprocal fishing relationships between Bunwurrung women and Baytayil—remain comparatively understudied. These areas of divergence highlight opportunities for future collaborative research, demonstrating potential for Bunurong Knowledge to inform new directions for marine mammal research and conservation.

Wurnmarrinh is highlighted as a critical habitat region for Baytayil, with Stories describing it as a migratory stopover for Mother whales and Djudju to rest on their journey to warmer waters and the beaching of whales, offering a time of ceremony and an opportunity to connect with ancestors and keep the ocean in harmony. Despite this importance, Wurnmarrinh has not yet been prioritised as an important marine mammal habitat, and as such is understudied.

Descriptions of Baytayil travelling west carrying ancestors to **Karralk** (the place that waits in the west for those who leave us in death), often accompanied by their calves, likely relate to observations of humpback whales (and potentially southern right whales) on their southward migration. Although mother and calf pairs into the northern reaches of Wurnmarrinh are not frequently observed in the present day, this disparity represents a gap in knowledge regarding the importance of the Western Port Bay region for whales that journey through Victorian waters. There is potential for the habitat suitability and specific environmental factors that may support this behaviour to be investigated. Furthermore, the prioritisation of conservation management within the region could also be explored, with consideration for cultural connection to be further recognised.

Indigenous Knowledges may also contribute to reconstructing historical baselines for population abundance, distribution and habitat use. Brough et al. (2025) demonstrated that weaving historical and WS systems can provide complementary insights into pre‐colonial species occurrences. A key example of this identification is **Te Ākau** (Bream Bay) and surrounding areas as important habitat for **tohorā** (southern right whales), knowledge that could not have been generated through conventional scientific surveys, as tohorā have been extirpated from the region due to industrial whaling. Despite their current absence, mātauranga Māori identified the region as an important wintering ground for the species prior to large‐scale exploitation.

This study illustrates the potential for IEK and WS methodologies to contribute complementary understanding of ecological function, species distributions and habitat use. Similarly, the Knowledge contained within these Bunurong Stories may provide valuable insights into historical marine mammal distributions and associations with Naarm and Wurnmarrinh that are not yet captured by contemporary datasets. The habitat usage and movement patterns described within the Stories may reflect ecological conditions that supported historical populations, identify habitats that remain important to contemporary populations or highlight areas that may facilitate future population recovery. Therefore, these Stories provide not only a record of long‐term ecological observations but also a potential foundation for guiding future research and conservation initiatives.

#### 4.4.3 Implications for Conservation, Management, Knowledge Transmission

While the documentation and sharing of these Bunurong Stories, as told by AGD, represents the initial stages of bringing IEK and WS together within marine research, it also demonstrates the value of culturally informed and collaborative approaches to research. The integration, weaving and braiding of knowledge systems has been successfully applied across a range of Australian and international contexts (Kennett et al. 2004; Preuss and Dixon 2012; Moore and Hauser 2019; Brough et al. 2025), supporting more holistic understandings for environmental management. This approach also aligns with the Australian Strategy for Nature (DCCEEW 2024), which aims to engage with Traditional Owners throughout all sectors to use WS and IEK to inform best‐practice management of marine and terrestrial environments.

Within Australia, opportunities exist to further support Indigenous‐led marine conservation and management through Sea Country Indigenous Protected Areas, (‘Sea Country Indigenous Protected Areas Program Grants ‐ DCCEEW’ n.d.), Sea Country Ranger Programs and collaborative marine monitoring programs. The establishment and expansion of these initiatives may facilitate the protection of habitats that are both ecologically and culturally significant, while also supporting Indigenous self‐determination in environmental and resource management (Reed et al. 2021). These approaches also recognise that ecosystem recovery extends beyond ecological restoration and should also involve the restoration of cultural relationships, responsibilities, ceremonial practices and connections to Sea Country. Such initiatives are already underway occurring on Bunurong Country in Milawul, where BLCAC have established partnerships with Monash University and Phillip Island Nature Parks to investigate Seal Rocks, Australia's largest Australian fur seal colony. This collaboration aims to improve the understanding of climate change on critical marine habitat, while recognising and protecting its deep Bunurong Cultural Values (BLCAC 2024). At a national level, this study provides an example of how IEK can be respectfully engaged within marine research, and potentially conservation. By exploring areas of convergence between yulendji Bunwurrung and WS, the study highlights opportunities for collaboration, knowledge sharing and the development of future research questions across Australia's marine and coastal environments and self‐determined management of Sea Country.

As evidenced by the **Miyapunu** (marine turtle) partnerships between the Dhimurru Land Management Aboriginal Corporation (Dhimurru) and Yolŋu Tradtional Owners, collaborative approaches can successfully bring IEK and WS together in environmental management and conservation research (Kennett et al. 2004). This project saw relationships established between Dhimurru, Charles Darwin University and the Northern Territory Parks and Wildlife Service to investigate marine turtle distribution and abundance, and to quantify Indigenous harvesting of eggs and turtles. Through a genuine ‘two‐way’ ganma learning and management approach, the project considered both ecological sustainability and cultural responsibilities. Importantly, the partnership recognised the Yolŋu peoples' responsibilities for caring for Country and positioned IEK as a foundation of research rather than simply incorporating Indigenous participation into pre‐existing WS goals. Similarly, this present study seeks to contribute to the early stages of cross‐cultural collaboration as outlined by (Ens et al. 2012) by recognising the validity of both Indigenous and non‐indigenous environmental philosophies and creating more opportunities for improved cross‐cultural understanding, respect and collaboration. It is hoped that future research and monitoring initiatives arising from this work can continue to develop through the framework of Walagunh Baahnbula, supporting meaningful integration of Bunurong Knowledge and WS in marine mammal conservation, management and cultural recognition.

Despite these opportunities, progress towards fulfilling UNIDRIP commitments within Australia remains uneven (Conrad et al. 2025). The inclusion of Indigenous languages in ocean governance and conservation research has been identified as one mechanism to improve equity and access for Indigenous communities (Conrad et al. 2025). However, the legacy of assimilationist policies and the diversity of Australian Indigenous languages (over 250 languages identified) present ongoing challenges (Wigglesworth and Keegan 2013). Within the context of this study, the incorporation of Bunwurrung language represents a small but meaningful contribution towards increasing language visibility in marine ecological research and formal scientific literature. Integrating local languages into education, ocean literacy initiatives and place‐based environmental management may also support broader recognition of Indigenous knowledge systems and relationships with Sea Country. Language plays a particularly important role in environmental stewardship, acting as a medium through which people connect with Sea Country and Land (Ferguson and Weaselboy 2020). Furthermore, the inclusion of Bunurong artwork in this study sought to incorporate Indigenous communication practices within the research process and dissemination of findings. As a form of visual Storytelling, artwork can support the sharing of Knowledge across cultural gaps and into ecological research and may facilitate engagement with both Indigenous and non‐Indigenous communities (Zurba and Berkes 2014).

At a local level, this study aims to support the objectives of the Bunurong Land Council Aboriginal Corporation to research, manage and protect flora and fauna of spiritual, ecological, cultural, and customary significance (The Rule Book of Bunurong Land Council (Aboriginal Corporation) ICN:3630 2026). By documenting and sharing Bunurong Stories within an ecological context, this work contributes to the preservation, transmission and continued application of Knowledge, while also highlighting the inclusion of holistic cultural values in nature conservation and demonstrating the potential for IEK to inform future marine mammal research and management across Bunurong Sea Country.

### 4.5 Challenges and Considerations of Cross‐Cultural Research

Several studies have shown the value of recognising the co‐existence and complementarity of multiple knowledge systems (Andreotti et al. 2011; Reid et al. 2021; Stein et al. 2024), rather than positioning IEK and WS as competing or requiring assimilation. However, efforts to engage with IEK (and sciences) do not always unfold in straightforward and generative ways. As Hunt (2014) notes, non‐Indigenous researchers seeking to engage with IEK must navigate a process that is rarely predictable, linear or easily accommodated in Western frameworks. Challenges may arise when attempting to bridge the relational nature of IEK sharing with the timelines, outputs and expectations that are often associated with Western research and Western‐based constraints.

Messiness is also a term that has been used to summarise the epistemic and methodological challenges of meaningfully working across knowledge systems (Gould et al. 2025). Building respectful relationships with participants, ensuring there is space for relationship building and establishing a rapport before moving forward with the research are essential components of ethical cross‐cultural research (Cooke et al. 2022), yet these processes require time and flexibility that may not always align with institutional or funding body research timelines. While collaborative approaches have the potential to support more equitable environmental management, Indigenous Peoples continue to experience disempowerment within many governance and decision‐making processes concerning Country (‘Governance | Australia State of the Environment 2021’, n.d.). Furthermore, the failure to establish respectful relationships, alongside the ongoing under‐recognition and exclusion of IEK in environmental management and research, may contribute to ineffective decision‐making and the continued mismanagement of Country.

These considerations were important throughout this study and informed both the development of relationships with BLCAC and Bunurong Knowledge holders, as well as the collaborative interpretation of the Stories that were presented. While the building of respectful relationships remained central to this study, the project nevertheless encountered challenges in working within conventional Western‐based institutional and funding body timelines. Based on this experience, we recommend that cross‐cultural research projects should incorporate adequate time for relationship building, ethics applications and collaborative engagement during the project planning and funding stages. Embedding these essential components of cross‐cultural research in project timelines will better support ethical, respectful and culturally appropriate partnerships.

## 5 Conclusion

The inclusion of holistic cultural values and IEK in environmental conservation and facilitating the sharing of IEK and Stories is a timely and important task (Robin et al. 2022). Through collaborative analysis of Bunurong Stories shared by Bunurong Elder, Aunty Gail Dawson, this study shared ecological insights relating to marine mammal behaviour, movement patterns, habitat use, social structures, and historical changes, showcasing how IEK can mix with and complement contemporary ecological research. These Stories demonstrated areas of convergence with contemporary marine mammal science while also identifying habitat regions, ecological relationships and interspecific relationships that remain underrepresented within scientific literature. Collectively, these findings highlight the potential for Bunurong Knowledge to contribute to marine mammal ecology, generate new research questions and support future collaborative approaches to conservation management. Recognising and engaging with Bunurong Knowledge through Walagunh Baahnbula underscores the value of meaningful partnerships with Traditional Custodians in advancing understanding of Warrinh and Biik and sharing Knowledge of the marine mammals within Bunurong Sea Country.

As told by Aunty Gail Dawson:

We keep telling our sea‐women stories when we are all sitting down doing gentle work, or tracing the stories in the night sky, or walking on a sea‐woman songline. We know that no matter how we are hunted and killed, by telling our stories, we keep safe the knowledge that keeps Country and all it holds safe and balanced.

## Author Contributions

**Jemima Beddoe:** conceptualization (equal), data curation (equal), funding acquisition (equal), investigation (lead), writing – original draft (equal). **Gail Kunwarra Dawson:** conceptualization (equal), data curation (equal), writing – original draft (equal), writing – review and editing (equal). **Jeff Shimeta:** conceptualization (equal), funding acquisition (equal), supervision (equal), writing – review and editing (equal). **Kate Robb:** conceptualization (equal), funding acquisition (equal), supervision (equal), writing – review and editing (equal).

## Funding

This work was supported by RMIT University (PRJ00002514), Department of Energy, Environment and Climate Action (OPP‐75996), and an Australian Government Research Training Program (RTP) Scholarship.

## Disclosure

Positionality statement: This work was a collaboration between Indigenous Knowledge holders and conservation scientists. The non‐Indigenous researchers on the authorship team acknowledge Country, in particular Bunurong Country, where we have had the privilege of undertaking this research. We pay our respects to First Nation peoples, including Elders past and present. We collectively express our gratitude to the Bunurong Land Council Aboriginal Corporation, Aunty Gail Dawson and Jayde Halls for sharing their Knowledge and collaborating with us.

## Ethics Statement

Ethical approval for this study was obtained from the RMIT University Human Ethics Committee (Approval number: 27507).

## Conflicts of Interest

The authors declare no conflicts of interest.

## Data Availability

The authors confirm that the data supporting the findings of this study are available within the article.
